# Effects of Shenfu Injection in the Treatment of Septic Shock Patients: A Multicenter, Controlled, Randomized, Open-Label Trial

**DOI:** 10.1155/2016/2565169

**Published:** 2016-06-30

**Authors:** Yi Li, Xinchao Zhang, Peihong Lin, Haibo Qiu, Jie Wei, Yu Cao, Shuming Pan, Joseph Walline, Chuanyun Qian, Zhigang Shan, XueZhong Yu

**Affiliations:** ^1^Emergency Department, Peking Union Medical College Hospital, Beijing 100730, China; ^2^Emergency Department, Beijing Hospital of the Ministry of Health, Beijing 100005, China; ^3^Emergency Department, The First Affiliated Hospital of Fujian Medical University, Fuzhou 350005, China; ^4^Emergency Department, Zhongda Hospital, Dongnan University, Nanjing 210009, China; ^5^Emergency Department, Hubei Provincial Hospital, Wuhan 430000, China; ^6^Emergency Department, The First Affiliated Hospital of Huaxi Medical College of Sichuan University, Chengdu 610044, China; ^7^Emergency Department, Xinhua Hospital, Shanghai Jiao Tong University, Shanghai 200093, China; ^8^Emergency Department, Saint Louis University, Saint Louis, MO 63130, USA; ^9^Emergency Department, The First Affiliated Hospital of Kunming Medical University, Kunming 650034, China; ^10^Emergency Department, PLA 263 Hospital, Beijing 101199, China

## Abstract

The effect of Shenfu on biochemical parameters and survival during resuscitation in patients with septic shock was examined. This was a multicenter, controlled, randomized, open-label trial carried out in 210 patients with septic shock from seven medical centers in China. They were randomized to Shenfu or saline. The primary outcome was lactate clearance. The secondary outcomes were shock index normalization, dose of vasopressors, ICU stay, hospital stay, and mortality. A total of 199 patients completed the trial. Blood pressure, heart rate, and other routine lab tests showed no difference between the groups. Lactate levels and lactate clearance were similar between the two groups. Hospital and ICU stay were similar between the two groups. When considering all patients, the 7- and 28-day mortality were similar between the two groups, but when considering only patients with lactate levels ≥4.5 mmol/L, the Shenfu group showed a better 7-day survival than the control group (7 days: 83.3% versus 54.5%, *P* = 0.034; 28 days: 72.7% versus 47.6%, *P* = 0.092). Shenfu may improve the 7-day survival in patients with impaired lactate clearance (≥4.5 mmol/L), but the mechanism for this effect is unclear. Additional studies are necessary to characterize the hemodynamic changes after Shenfu infusion. This trial is registered with ChiCTR-TRC-11001369.

## 1. Introduction

Septic shock is a very common illness encountered in emergency departments (EDs) and intensive care units (ICUs), representing 10% of all ICU admissions [1]. Mortality from septic shock is still high despite the best available treatments (18–35%) [2]. As such, improving the survival rate of patients with septic shock is an important area of study [3]. Traditional treatments for septic shock include broad spectrum antibiotics, volume resuscitation, and vasopressor therapy; transfusion, glucose control, renal replacement therapy, thrombosis prophylaxis, and nutritional support can also be considered according to the patient's condition [3].

Traditional Chinese Medicine (TCM) goes back over 1000 years, and it remains one of the main types of therapy in China [4]. In 2006, TCM health care facilities and practitioners provided care for over 200 million outpatients and some 7 million inpatients in China, accounting for 10–20% of health care in China [5]. However, the literature in TCM has been hampered by the low methodological quality of TCM clinical trials, publications in Chinese not being included in systematic reviews, and uncertainty regarding the safety of various herbs, which, taken together, inhibit the spread of TCM to other countries [6].

Herbal intravenous injections are the most common type of TCM used in ICUs in China [7]. “Shenfu” is an injectable herbal medicine and has been widely used in China for the treatment of septic shock [8]. Shenfu injection is a modern Chinese medicine preparation derived from a traditional formulation called the “Shenfu decoction.” It is prepared from red ginseng (steamed roots of* Panax ginseng*) and aconite (processed lateral roots of* Aconitum carmichaelii*). Aconite alkaloids and ginsenosides are the main active ingredients in Shenfu, and modern pharmacological evaluations have demonstrated that ginsenosides are the determinant contributor to the vasodilator benefit of Shenfu, whereas the alkaloids play a vital role in the cardiac electrophysiological effect of Shenfu by blocking ion channels [9]. Shenfu injection is frequently used in EDs and ICUs in China and has been shown to have some beneficial effects in rescuing patients in septic shock [10].

Our recently published meta-analysis has evaluated the results of available randomized controlled trials (RCTs) using Shenfu as compared with conventional therapies [11]. Only 12 RCTs published in Chinese involving 904 participants were identified. These studies suggest that Shenfu can increase blood pressure and decrease the heart rate. Seven of these studies suggest a lower mortality in the Shenfu group compared with conventional therapies. Nevertheless, most of these previous studies in patients in septic shock lack clear randomization definition, had small sample sizes, and were carried out at a single center, and only a few were rigorously controlled studies.

Therefore, there is a need for a high-quality RCT of Shenfu injection in critical care settings to explore its impact on mortality and other rescue parameters. The aim of this study was to examine the effect of Shenfu on biochemical parameters and survival during resuscitation in patients with septic shock. We hypothesized that Shenfu may increase blood pressure and the oxygen extraction fraction, decrease lactate levels, increase lactate clearance rates, and improve the survival rate compared to the control group.

## 2. Materials and Methods

### 2.1. Patients

This study included patients from seven university tertiary hospitals treated between May 1, 2011, and December 31, 2012. Septic shock was diagnosed according to guidelines at the time of study [12]. The inclusion criteria were (1) diagnosis of septic shock and (2) age 18–70. The exclusion criteria were (1) very poor prognosis (<24 h); (2) cardiopulmonary resuscitation; (3) late-stage malignant tumors; (4) history of coronary artery disease; (5) history of allergies; (6) active “do not resuscitate” (DNR) order; (7) pregnant or lactating women; (8) being enrolled in another clinical trial in the last three months; (9) requirement for hemopurification during the observation time; or (10) any other special conditions judged by the clinical staff at the time of enrollment to preclude participation. Patients were removed from the study after enrollment if a misdiagnosis of septic shock was found or if some prohibited drugs were used. Patients were considered as dropouts when (1) they left the ICU against medical counsel; (2) they have inability to complete follow-up; or (3) they have poor compliance. The reasons for dropping out patients were recorded.

### 2.2. Study Design

This was a multicenter, controlled, randomized, open-label trial carried out in patients with septic shock from seven university medical centers, led by the Peking Union Medical College Hospital. This trial was approved by the Ethics Committee of the Peking Union Medical College Hospital on May 30, 2011 (Study # S-377), and was registered (ChiCTR-TRC-11001369). A written informed consent was obtained from the patient or their legal representative.

### 2.3. Randomization

Patients were randomized centrally according to age, gender, and shock index. The age of enrollees was stratified into three levels: 18–45, 46–60, and 61–70. The shock index was stratified as >1 but <1.5, >1.5 but <2, and >2. The allocation ratio for shock index was 1 : 1 : 1. Randomization codes were assigned strictly sequentially by the central principal investigator as patients became eligible for randomization. The randomization codes were prepared by a statistician using random number tables generated using SAS 9.3 (SAS Institute, Cary, NY, USA). Eligible patients were randomized to the Shenfu treatment group or the control group in a 1 : 1 ratio. There was no blinding.

### 2.4. Treatments

Routine treatment of septic shock was performed as recommended by current guidelines at the time of the study [12]. The Shenfu group received routine treatments and Shenfu injection (Ya'an Sanjiu Pharmaceutical Co., Ltd., Sichuan, China; 30 mL/h for 3 h for the first day; from the second day after enrollment, 100 mL of Shenfu was diluted in 200 mL of normal saline and infused within two hours, once a day, for five days). The control group received routine treatment (identical to the Shenfu group) and normal saline infusion according to the exact same administration scheme as in the Shenfu group.

### 2.5. Clinical Variables

Vital signs were recorded every hour for the first 24 h, then every 4 h for 24 h, and once a day thereafter. Complete blood count (CBC), routine urinalysis, and stool testing were done. Alanine transaminase (ALT), aspartate aminotransferase (AST), blood urea nitrogen (BUN), and creatinine (Cr) were tested. An electrocardiogram (ECG) was recorded for every patient. These tests were done at admission and 24 and 48 h after admission. The infectious focus and etiology were recorded for each patient. The shock index was calculated at admission. Acute physiology and chronic health evaluation II (APACHE II) and Glasgow coma score (GCS) were calculated and recorded. Blood was collected to test for arterial lactate, as well as serum glucose, sodium, and potassium levels. Coagulation tests, platelet count, prothrombin time (PT), activated partial thromboplastin time (aPTT), fibrinogen, troponin I, creatine kinase (CK), and CK-MB were recorded. Arterial lactate was tested on admission, then after 3, 6, 12, 24, 36, and 48 h, and then every day. Central venous pressure (CVP) was measured every three hours in the first six hours after admission and then every six hours for two days.

### 2.6. Outcomes

The primary outcome was lactate clearance. The secondary outcomes were the time needed for shock index normalization, doses of vasopressors (dopamine and norepinephrine), 7- and 28-day mortality, hospital stay, and ICU stay.

### 2.7. Safety

Side effects were recorded if they occurred (e.g., tachycardia, rashes, dyspnea, dizziness, headaches, nausea and vomiting, and tremors).

### 2.8. Follow-Up

The patients were followed up for 28 days either by medical visit or by telephone.

### 2.9. Statistical Analysis

We collected data using an Electronic Data Capture System [the Data Analysis System (DAS) for Electronic Data Capture (EDC)]. With this system, data checking was done automatically, all data can be traced back, and operation tracks were kept. Because this was the first rigorous RCT of Shenfu, the sample size could not be calculated and 200 septic shock patients (100/group) were enrolled. Efficacy and safety analyses were performed on an intent-to-treat (ITT) basis. Efficacy was determined using the full analysis set (FAS; all patients who did not drop out), while safety was determined using the safety set (SS; all patients who received at least one dose of Shenfu). Continuous data are presented as mean ± standard deviation and were analyzed using the Student *t*-test. Categorical variables are presented as frequencies and were analyzed using the chi-square test. Analyses were conducted using SAS 9.3. (SAS Institute, Cary, NY, USA). Two-sided *P* values <0.05 were considered statistically significant.

## 3. Results

### 3.1. Recruitment

**Figure 1:**
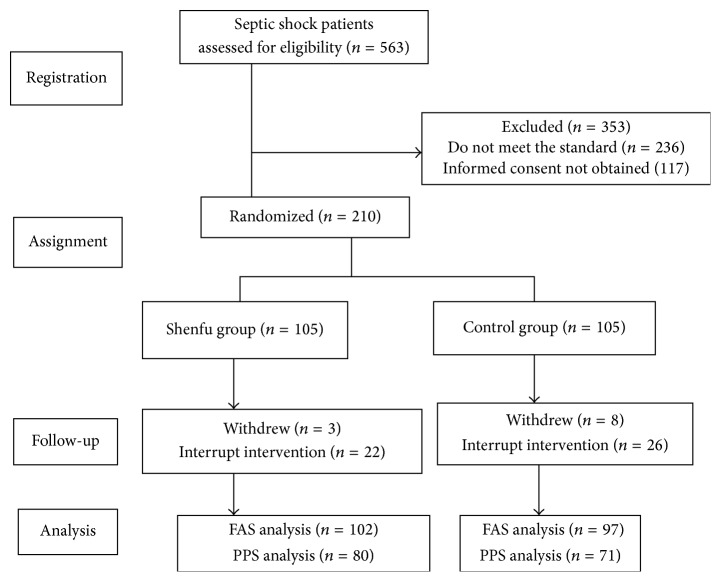
Study flowchart.

This trial enrolled its first patient on July 5, 2011, and finished enrollment on June 6, 2014. Once the objective sample size of 200 was reached, the enrollment was stopped. A total of 563 suspected septic shock patients were admitted during the study period; 210 patients were enrolled and 199 finished the trial. The study group included 124 females and 75 males; 102 patients were allocated to the Shenfu group and 97 to the control group.  Figure 1 presents the study flowchart. Seven patients were removed from the study because arterial lactate was not detected, and four cases withdrew their consent after enrollment (two from each group).

### 3.2. Characteristics of the Patients

There were no differences in all the baseline data between the two groups, including the infection sites (Table 1). After 6 days of treatment, compared with controls, patients in the Shenfu group showed lower red blood cell count (*P* = 0.01), higher CVP (*P* = 0.02), and higher ScvO_2_ (*P* = 0.02) (Table 2). All other parameters were similar between the two groups.

### 3.3. Primary Outcome

**Figure 2:**
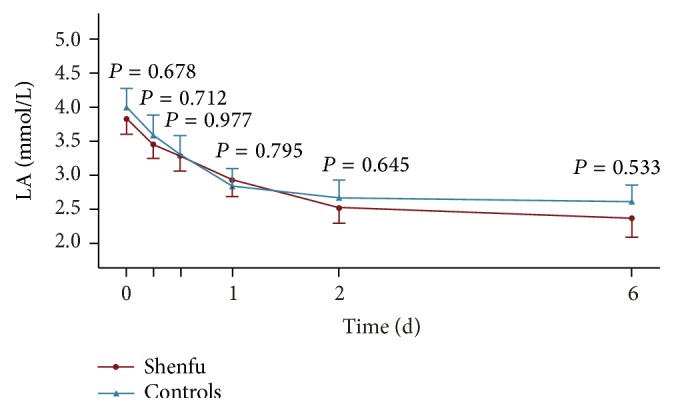
The kinetic change of arterial lactate from 0 to 6 days (mean ± SE, Shenfu = 102; control = 97).

There were no differences between the two groups when considering the primary outcome that is arterial lactate and its clearance at each observational time point (Table 3). The kinetic change of lactate in the first 6 days was shown in Figure 2. *P* value in each six-day time point showed that there was no statistical difference between the two groups.

No matter 24 h lactate clearance rate was larger than 10% or not, the difference between the Shenfu and control group was not significant, according to the results of logistic regression analysis, in which adjusted variables APACHE II scores, the infection site, and the baseline level of lactate were analyzed.

### 3.4. Secondary Outcomes

There were no differences between the two groups when considering the secondary outcomes. The 7-day survival was similar between the two groups (Shenfu: 82.7% versus controls: 82.6%, *P* = 0.994) as well as the 28-day survival (Shenfu: 72.0% versus controls: 69.9%, *P* = 0.086) (Table 4).

Patients were stratified according to arterial lactate for the ancillary analysis, and the cutoffs of 4.0, 4.5, and 5.0 mmol/L were selected based on previous studies [13–15]. When using the 4.5 mmol/L cutoff point, a difference was observed in the 7-day survival (Shenfu: 83.3% versus controls: 54.5%, *P* = 0.034) (Table 4).

**Figure 3:**
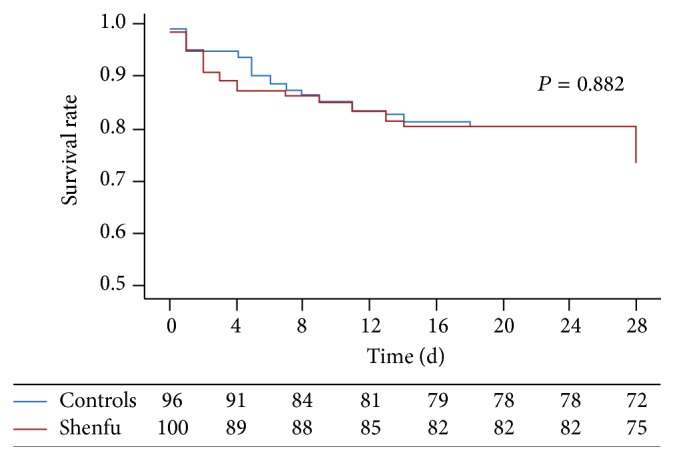
Kaplan-Meier survival curves for all patients. The blue line is the survival rate of the control group, while the red line is the survival rate of the Shenfu group.

**Figure 4:**
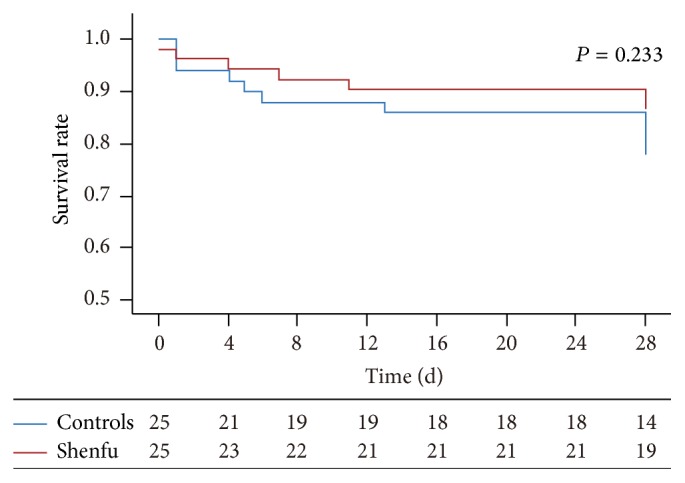
Kaplan-Meier survival curves among the patients with baseline arterial lactate >4.5 mmol/L.

The death hazard ratio (Shenfu versus controls) was 1.042 (95% CI 0.605~1.795), which means that Shenfu would not reduce the total risk of death compared with the controls group. The Kaplan-Meier survival curves were provided in Figures 3 and 4. In Figure 3, the 28-day survival curves of the two groups were overlapped and no statistical differences were found (*P* = 0.882). In Figure 4, the survival curves of the two groups with lactate over 4.5 mmol/L were separated obviously, but no statistical difference was found (*P* = 0.233).

The 25th percent survival time was 28 days for two groups for all population. However, in the subgroup of patients whose baseline level of lactate was more than 4.5 mmol/L, the 25th percent survival time of the Shenfu group was much longer than control group (28 days versus 13 days).

### 3.5. Safety

Side effects (pruritus) were found in only one patient in the Shenfu group, and it was determined as not being related to the study drug.

## 4. Discussion

Shenfu injection has been used for a long time in China to treat septic shock, but there is a lack of RCTs about its efficacy [11]. Therefore, this study aimed to examine the effects of Shenfu on biochemical parameters and survival during resuscitation in patients with septic shock. Results showed that there were no differences in blood pressure, heart rate, and other routine lab tests between the two groups. Lactate levels and lactate clearance were similar between the two groups. When considering all patients, the 7- and 28-day mortality were similar between the two groups, but when considering only patients with lactate levels ≥4.5 mmol/L, the Shenfu group showed a better 7-day survival than the control group.

Shenfu injection is a well-known TCM and has won worldwide attention. A. Varon and J. Varon [16] believe that Shenfu injection sounds promising for the early treatment of sepsis patients. Therefore, new research efforts should be carried on about Shenfu in order to elucidate its mechanisms.

Lactate is an important marker for the severity of septic shock and sepsis and is positively correlated with disease severity. Levy et al. [17] reported that higher lactate levels were associated with more severe septic states. They also noted that the mortality of septic patients was the highest (46.1%) in patients with combined hypotension and lactate ≥4 mmol/L compared with hypotension alone (36.7%) or lactate over ≥4 mmol/L alone (30%). Indeed, patients having lactate levels ≥4 mmol/L are thought of as being more severe septic patients [18, 19]. Lactate clearance is another important marker in septic patients, and higher lactate clearance is associated with better outcomes [20, 21].

The primary outcome of this trial was lactate clearance, for which there were no statistical differences between the two groups. The arterial lactate at each of the six time points (0 hours, 6 hours, 12 hours, 24 hours, 48 hours, and 6 days) showed no difference either. Lactate had been examined in seven previous studies about Shenfu injection in septic shock as an outcome measure [8, 10, 22–26]. The pooled lactate levels in these seven studies showed no differences after one hour of treatment but showed significantly lower lactate levels after 6 and 24 hours of treatment in the Shenfu groups. Lactate levels have been compared after 1, 6, and 24 hours of treatment in 3, 6, and 3 studies, respectively [11]. Compared with conventional therapy, Shenfu was found to increase the mean arterial pressure after 1 hour, normalize heart rate after 6 hours, and lower serum lactate levels after 6 and 24 hours [11].

In this study, there was no difference in survival when considering the whole group of patients. Seven previous studies reported mortality data after Shenfu treatment [11]. The mortality in the Shenfu group was significantly decreased compared with that of the control group in six of the seven studies [11]. Indeed, the reported mortality varied from 3.1% (1/32) versus 9.4% (3/32) in the study by He et al. [10] to 56.5% (26/46) versus 64.4% (29/45) in the study by Dong and Shen [8]. Therefore, the wide variability in mortality among these studies is one of the main factors prohibiting the acceptance of these results. In addition, there was no improvement in 28-day mortality in the study by Dong and Shen [8] carried out in 48 patients in the Shenfu group and 45 controls. Nevertheless, the mortality rates observed by Dong and Shen [8] were comparable to those of the present study. Another recent study showed no difference in mortality between the Shenfu group and controls [27].

However, previous studies about Shenfu in septic shock did not explore the survival outcome among subgroups stratified by lactate levels [11, 27]. Therefore, to investigate the mortality in some specific groups, the patients were stratified based on different cutoffs for lactate: 4, 4.5, and 5 mmol/L [13–15]. Using 4.5 mmol/L, the mortality in the Shenfu group with lactate levels ≥4.5 mmol/L was significantly lower on day seven compared with controls (*P* = 0.034), but not on day 28. No statistical difference in the Kaplan-Meier 28-day survival curves of patients with arterial lactate ≥4.5 mmol/L was found, which seems contradictory with the result of the mortality rate. This study does not allow determining the exact reasons for this. The first reason may be the small sample size of the subgroups. Another reason is the difference of the two parameters: the mortality shows the actual death rate of the two groups in each time point (including the 7 days and the 28 days, the former has statistical difference, while the latter is without), while the Kaplan-Meier survival curves work for the predicted survival of 28 days.

Only two hemodynamic parameters were statistically different between the two groups: CVP and ScvO_2_ were higher in the Shenfu group than in controls. It seems that Shenfu might have the ability of improving the microcirculation. Indeed, the use of Shenfu in patients with cardiac arrest was shown to increase cardiac output (CO) [9]. One of the main ingredients in Shenfu is “Fuzhi,” which has been shown in animals and clinical trials to improve cardiac function [9]. It was found in a swine model of cardiac arrest that Shenfu treatment produced better left ventricular systolic function, CO, and ejection fraction after restoration of spontaneous circulation (ROSC).

In a study by Li et al. [27], mean arterial pressure, cardiac index, and systemic vascular resistance index were increased by Shenfu, while lactate and heart rate were decreased [27]. In this study, there were no differences in MAP or heart rate. The impact of Shenfu on heart rate, blood pressure, CO, and other hemodynamic changes needs to be explored further in future trials.

There are few clinical studies about the molecular mechanisms of Shenfu in patients, but some animal studies may provide clues about the mechanisms of Shenfu on the molecular level. Data from swine models showed that Shenfu can alleviate the cardiac dysfunction after cardiac arrest by improving energy delivery and alleviation of oxygenation and lipid peroxidation; in addition, there is some additional evidence that Shenfu can inhibit the expression of Bcl-2, Bax, and caspase-3, decrease apoptosis, alleviate injuries to the heart [28], decrease cardiac *β*-adrenergic receptor sensitivity [29], and be generally cardioprotective. Shenfu has also been shown to inhibit apoptosis in lung tissue and improve antilipid peroxidation after cardiac arrest [30]. In addition, Shenfu can improve the immune function in the lungs [31]. Shenfu can decrease the expression of TNF-*α*, block malignant inflammation, improve the microcirculation, and extend the time of tissue and cellular viability without oxygen [32]. In addition, Shenfu may improve recovery of immunologic function by balancing cytokine secretion [33]. Shenfu has been shown in rats to decrease injury to the central nervous system after cerebral ischemia by decreasing calcium overload, suppressing inflammation, and decreasing apoptosis [34]. In addition, Shenfu can protect the brain after resuscitation by stabilizing the cellular membrane, suppressing the opening of the mitochondrial permeability transition pore, and alleviating the dysfunction of cerebral mitochondria [35]. Nevertheless, these mechanistic data are exploratory at best and additional studies are necessary to address this issue.

This study is not without limitations.

We have not chosen the blinded design because of the following reason: (1) objective parameters, such as the mortality and the lactate clearance, were chosen as the outcome, not the subjective parameters; so our result may be influenced less by the design; (2) it is difficult to make a blank injection in blind trial because the Shenfu injection is in yellow color; (3) the blinded label is expected to be open more often in the critical patients than patients with stable vital signs. We will design a blinded RCT in the next stage.

Indeed, no power analysis could be performed and it is a possibility that the sample size had been too small. No comprehensive study of biomarkers or mRNA/protein expression was carried out, and additional studies are necessary to understand the action mechanisms of Shenfu. In this pilot study, the mortality difference was found only in the subgroup of patients with lactate levels ≥4.5 mmol/L, and the number of patients in this subgroup was 45 (Shenfu 23 and normal saline 22), which might be too small to draw any firm conclusions. Hemodynamic parameters need to be observed together with mortality. Side effects are one of the main concerns regarding this herbal medication, and additional clinical studies are needed to explore the use of Shenfu.

## 5. Conclusions

Shenfu may improve the 7-day survival in patients with impaired lactate clearance (≥4.5 mmol/L), but the mechanism for this effect is unclear. Additional studies are necessary to characterize the hemodynamic changes after Shenfu infusion.

## Figures and Tables

**Table 1:** Baseline characteristics of the patients.

	Shenfu (*n* = 102)	Controls (*n* = 97)	*P* value
Age (years)	54.0 ± 16.9	54.0 ± 16.9	0.979
Gender (male/female)	64/38	60/37	0.897
SBP (mmHg)	87.4 ± 19.1	84.0 ± 17.9	0.204
DBP (mmHg)	53.2 ± 13.8	52.0 ± 11.9	0.528
MBP (mmHg)	64.6 ± 14.7	62.7 ± 12.4	0.332
Shock index	1.4 ± 0.4	1.4 ± 0.3	0.407
Heart rate (bpm)	114.3 ± 19.8	111.5 ± 22.2	0.347
Respiratory rate (bpm)	23.2 ± 6.2	23.9 ± 5.5	0.412
Infection site^*∗*^ [*n* (%)]			0.493
Unknown origin of infection	28 (27.5)	40 (41.2)	
Pulmonary	37 (36.3)	30 (30.9)	
Multiple	9 (8.8)	7 (7.2)	
Peritoneal	6 (5.9)	3 (3.1)	
Urinary	4 (3.9)	4 (4.1)	
Catheter related	5 (4.9)	2 (2.1)	
Others	13 (12.7)	11 (11.3)	

Notes: ^*∗*^ *R* × *C* chi-square test. Multiple means that there were more than two sites of infection.

SBP: systolic blood pressure; DBP: diastolic blood pressure; MBP: mean blood pressure.

**Table 2:** Clinical indexes during treatment.

	Shenfu	Controls	*P* value
*48 h*	*n* = 102	*n* = 97	
Blood glucose (mmol/L)	9.01 ± 6.33	7.81 ± 3.07	0.134
PT (s)	14.20 ± 3.63	14.39 ± 7.51	0.858
APTT (s)	37.28 ± 9.00	36.76 ± 9.81	0.749
Fibrinogen (g/dL)	3.76 ± 1.64	4.60 ± 6.12	0.304
BUN	10.44 ± 7.86	9.28 ± 7.10	0.346
Cr	101.16 ± 71.98	104.94 ± 94.68	0.784
ALT	78.51 ± 140.56	54.96 ± 54.54	0.165
AST	102.29 ± 161.02	74.46 ± 91.01	0.235
WBC	13.33 ± 9.61	12.06 ± 10.22	0.422
RBC	3.40 ± 0.68	3.75 ± 1.01	0.012
HCT	30.66 ± 6.28	31.71 ± 7.31	0.331
Platelets (×10^9^/L)	108.49 ± 75.42	115.31 ± 81.19	0.585

*48 h*	*n* = 15	*n* = 10	
CVP (cmH_2_O)	10.67 ± 4.46	6.35 ± 3.16	0.018
ScvO_2_	79.63 ± 13.28	54.70 ± 28.81	0.017

*6 d*	*n* = 102	*n* = 97	
Arterial lactate (mmol/L)	2.37 ± 2.81	2.60 ± 2.52	0.533
Lactate clearance	−0.40 ± 0.59	−0.33 ± 0.39	0.369

*24 h*	*n* = 102	*n* = 97	
APACHE 2	14.75 ± 9.13	15.14 ± 11.22	0.846

*5 d*	*n* = 102	*n* = 97	
Urine output (mL/h)	91.91 ± 49.97	102.18 ± 40.25	0.274

*6 d*	*n* = 57	*n* = 50	
Systolic pressure (mmHg)	33.88 ± 19.44	34.72 ± 25.97	0.851
Diastolic pressure (mmHg)	15.46 ± 16.25	13.12 ± 16.52	0.463
MAP (mmHg)	21.60 ± 15.82	20.32 ± 18.26	0.699
Heart rate (bpm)	−24.51 ± 21.78	−26.20 ± 26.74	0.719
Dose of dopamine (mg)	842.04 ± 835.81	1224.71 ± 1704.24	0.237
Number of dopamine uses	*n* = 29	*n* = 37	0.229
Dose of norepinephrine (mg)	72.67 ± 64.19	80.59 ± 137.68	0.759
Number of epinephrine uses	*n* = 36	*n* = 35	0.99
ICU day (day)	9.4 ± 11.3	9.1 ± 14.4	0.895
Total hospital day (day)	15.2 ± 13.7	14.1 ± 15.8	0.675

PT: prothrombin time; aPTT: activated partial prothrombin time; BUN: blood urea nitrogen; Cr: creatinine; ALT: alanine transaminase; AST: aspartate transaminase; WBC: white blood cells; RBC: red blood cells; HCT: hematocrit; CVP: central venous pressure; ScvO_2_: central venous oxygen saturation; APACHE: acute physiology and chronic health evaluation; ICU: intensive care unit.

**Table 3:** Kinetic changes of lactate and its clearance rate.

	Lactate in the Shenfu group (*N* = 102)	Lactate in the control group (*N* = 97)	Lactate clearance in the Shenfu group (*N* = 85)	Lactate clearance in the control group (*N* = 74)
0 h	3.83 ± 2.32	3.99 ± 2.87		
6 h	3.45 ± 2.19	3.59 ± 2.84	−0.07 ± 0.44	−0.10 ± 0.32
12 h	3.29 ± 2.24	3.30 ± 2.76	−0.13 ± 0.36	−0.15 ± 0.41
24 h	2.93 ± 2.40	2.83 ± 2.52	−0.22 ± 0.55	−0.28 ± 0.35
48 h	2.51 ± 2.28	2.67 ± 2.53	−0.34 ± 0.52	−0.33 ± 0.36
144 h	2.37 ± 2.81	2.60 ± 2.52	−0.40 ± 0.59	−0.33 ± 0.39

Lactate clearance was calculated and compared only in patients with elevated lactate. There was no difference at each time point between the two groups.

**Table 4:** Comparison of survival between the two groups.

	Shenfu *N* (%)	Controls *N* (%)	*P* value
*7 d*			
All patients mortality			
Alive	81 (82.7)	76 (82.6)	0.994
Dead	17 (17.3)	16 (17.4)	
Total (lost)	98 (4)	92 (5)	
Mortality of patients with arterial lactate ≥4.5 mmol/L			
Alive	20 (83.3)	12 (54.5)	0.034
Dead	4 (16.7)	10 (45.5)	
Total (lost)	24 (2)	22 (3)	
Mortality of patients with arterial lactate <4.5 mmol/L			
Alive	61 (82.4)	64 (91.4)	0.111
Dead	13 (17.6)	6 (8.6)	
Total (lost)	74 (2)	70 (2)	
*28 d*			
All patients mortality			
Alive	59 (72.0)	58 (69.9)	0.770
Dead	23 (28.0)	25 (30.1)	
Total (lost)	82 (20)	83 (14)	
Mortality of patients with arterial lactate ≥4.5 mmol/L			
Alive	16 (72.7)	10 (47.6)	0.092
Dead	6 (27.3)	11 (52.4)	
Total (lost)	22 (4)	21 (4)	
Mortality of patients with arterial lactate <4.5 mmol/L			
Alive	43 (71.7)	48 (77.4)	0.466
Dead	17 (28.3)	14 (22.6)	
Total (lost)	60 (16)	62 (10)	
