# Corrigendum to “Effects of Shenfu Injection in the Treatment of Septic Shock Patients: A Multicenter, Controlled, Randomized, Open-Label Trial”

**DOI:** 10.1155/2016/6460252

**Published:** 2016-10-12

**Authors:** Yi Li, Xinchao Zhang, Peihong Lin, Haibo Qiu, Jie Wei, Yu Cao, Shuming Pan, Joseph Walline, Chuanyun Qian, Zhigang Shan, XueZhong Yu

**Affiliations:** ^1^Emergency Department, Peking Union Medical College Hospital, Beijing 100730, China; ^2^Emergency Department, Beijing Hospital, National Center of Geriatrics, Beijing 100730, China; ^3^Emergency Department, The First Affiliated Hospital of Fujian Medical University, Fuzhou 350005, China; ^4^Department of Critical Care Medicine, Zhongda Hospital, School of Medicine, Southeast University, Nanjing 210009, China; ^5^Emergency Department, Renmin Hospital of Wuhan University, Wuhan 430060, China; ^6^Emergency Department, West China Hospital of Sichuan University, Chengdu 610041, China; ^7^Emergency Department, Xinhua Hospital, Shanghai Jiao Tong University School of Medicine, Shanghai 200092, China; ^8^Emergency Department, Saint Louis University, Saint Louis, MO 63110, USA; ^9^Emergency Department, The First Affiliated Hospital of Kunming Medical University, Kunming 650032, China; ^10^Emergency Department, PLA 263 Hospital, Beijing 101149, China

In the article titled “Effects of Shenfu Injection in the Treatment of Septic Shock Patients: A Multicenter, Controlled, Randomized, Open-Label Trial” [1], affiliations 2, 4, 5, 6, 7, 8, 9, and 10 were incorrect. The revised affiliations are shown above.

## References

[1] Li Y., Zhang X., Lin P. (2016). Effects of Shenfu injection in the treatment of septic shock patients: a multicenter, controlled, randomized, open-label trial. *Evidence-Based Complementary and Alternative Medicine*.

